# Severe allergic reactions after COVID-19 vaccination with the Pfizer/BioNTech vaccine in Great Britain and USA: Position statement of the German Allergy Societies: Medical Association of German Allergologists (AeDA), German Society for Allergology and Clinical Immunology (DGAKI) and Society for Pediatric Allergology and Environmental Medicine (GPA)

**DOI:** 10.1007/s40629-020-00160-4

**Published:** 2021-02-24

**Authors:** Ludger Klimek, Natalija Novak, Eckard Hamelmann, Thomas Werfel, Martin Wagenmann, Christian Taube, Andrea Bauer, Hans Merk, Uta Rabe, Kirsten Jung, Wolfgang Schlenter, Johannes Ring, Adam Chaker, Wolfgang Wehrmann, Sven Becker, Norbert Mülleneisen, Katja Nemat, Wolfgang Czech, Holger Wrede, Randolf Brehler, Thomas Fuchs, Thilo Jakob, Tobias Ankermann, Sebastian M. Schmidt, Michael Gerstlauer, Christian Vogelberg, Thomas Zuberbier, Karin Hartmann, Margitta Worm

**Affiliations:** 1 Center for Rhinology and Allergology Wiesbaden, Wiesbaden, Germany; 2 Clinic and Polyclinic for Dermatology and Allergology, University Hospital Bonn, Bonn, Germany; 3 Pediatric and Adolescent Medicine, Children’s Center Bethel, University Hospital OWL of Bielefeld University, Bielefeld, Germany; 4 Department of Dermatology, Allergology and Venereology, Hannover Medical School, Hannover, Germany; 5 Department of Ear, Nose and Throat Medicine, University Hospital Düsseldorf, Düsseldorf, Germany; 6 Clinic for Pneumology, University Medicine Essen-Ruhrlandklinik, West German Lung Center, Essen, Germany; 7 Clinic and Polyclinic for Dermatology, University Allergy Center, University Clinic Carl Gustav Carus, Technical University Dresden, Dresden, Germany; 8 Department of Dermatology and Allergology, RWTH Aachen, Aachen, Germany; 9 Clinic for Allergology, Johanniter Hospital in Fläming Treuenbrietzen GmbH, Treuenbrietzen, Germany; 10 Practice for Dermatology, Immunology and Allergology, Erfurt, Germany; 11 Medical Association of German Allergologists, Dreieich, Germany; 12 Skin and Laser Center at the Opera, Munich, Germany; 13 Ear, Nose and Throat Clinic, Klinikum rechts der Isar, Technical University of Munich, Munich, Germany; 14 Center for Allergy and Environment (ZAUM), Klinikum rechts der Isar, Technical University of Munich, Munich, Germany; 15 Dermatological Group Practice Wehrmann, Münster, Germany; 16 Ear, Nose and Throat Clinic, University of Tübingen, Tübingen, Germany; 17 Asthma and Allergy Center Leverkusen, Leverkusen, Germany; 18 Practice for Pediatric Pneumology/Allergology at the Children’s Center Dresden (Kid), Dresden, Germany; 19 Practice and Clinic for Allergology/Dermatology Schwarzwald-Baar Clinic, Villingen-Schwenningen, Germany; 20 Ear, Nose and Throat Specialist, Herford, Germany; 21 Department of Skin Diseases, Outpatient Clinic for Allergology, Occupational Dermatology and Environmental Medicine, Münster University Hospital, Münster, Germany; 22 Department of Dermatology, Venereology and Allergology, University Medical Center, Georg-August University, Göttingen, Germany; 23 Department of Dermatology and Allergology, University Hospital Gießen, UKGM Justus Liebig University Gießen, Gießen, Germany; 24 Clinic for Pediatric and Adolescent Medicine, Pneumology, Allergology, Neonatology, Intensive Care Medicine, Infectiology, Schleswig-Holstein University Medical Center, Kiel, Germany; 25 Center for Pediatric and Adolescent Medicine, Clinic and Polyclinic for Pediatric and Adolescent Medicine, University Medicine Greifswald, Greifswald, Germany; 26 Pediatric Pneumology/Pediatric Allergology, 2nd Clinic for Children and Adolescents, University Hospital Augsburg, Augsburg, Germany; 27 Department of Pediatric Pneumology/Allergology, Clinic and Polyclinic for Pediatric and Adolescent Medicine, Carl Gustav Carus University Hospital, Dresden, Germany; 28 Clinic for Dermatology, Venereology and Allergology, Charité—University Medicine Berlin, Berlin, Germany; 29 Clinic for Dermatology and Allergology, University Hospital Basel, Basel, Switzerland; 30 Allergologie und Immunologie, Klinik für Dermatologie, Venerologie und Allergologie, Charité – Universitätsmedizin Berlin, Campus Mitte, Charitéplatz 1, 10117 Berlin, Germany

**Keywords:** BNT162b2, Severe allergic reactions, Anaphylaxis, Vaccines, mRNA

## Abstract

Two employees of the National Health Service (NHS) in England developed severe allergic reactions following administration of BNT162b2 vaccine against COVID-19 (coronavirus disease 2019). The British SmPC for the BNT162b2 vaccine already includes reference to a contraindication for use in individuals who have had an allergic reaction to the vaccine or any of its components. As a precautionary measure, the Medicines and Healthcare products Regulatory Agency (MHRA) has issued interim guidance to the NHS not to vaccinate in principle in “patients with severe allergies”. Allergic reactions to vaccines are very rare, but vaccine components are known to cause allergic reactions. BNT162b2 is a vaccine based on an mRNA embedded in lipid nanoparticles and blended with other substances to enable its transport into the cells. In the pivotal phase III clinical trial, the BNT162b2 vaccine was generally well tolerated, but this large clinical trial, used to support vaccine approval by the MHRA and US Food and Drug Administration, excluded individuals with a “history of a severe adverse reaction related to the vaccine and/or a severe allergic reaction (e.g., anaphylaxis) to a component of the study medication”. Vaccines are recognized as one of the most effective public health interventions. This repeated administration of a foreign protein (antigen) necessitates a careful allergological history before each application and diagnostic clarification and a risk–benefit assessment before each injection. Severe allergic reactions to vaccines are rare but can be life-threatening, and it is prudent to raise awareness of this hazard among vaccination teams and to take adequate precautions while more experience is gained with this new vaccine.

## Background

On December 9, 2020, the Medicines and Healthcare products Regulatory Agency (MHRA) in the United Kingdom informed of severe allergic reactions in two employees of the National Health Service (NHS) in England following administration of BNT162b2 vaccine against COVID-19 (Coronavirus disease 2019). Both patients had a history of anaphylaxis, and as far as is currently known both recovered rapidly and completely from these severe allergic reactions. Since it is not clear which component of the vaccine triggered the reaction, an investigation was initiated to fully understand the two incidents and their causes.

The British summary of product characteristics (SmPC) for the BNT162b2 vaccine already includes reference to a contraindication for use in individuals who have had an allergic reaction to the vaccine or any of its components [1]. As a precautionary measure, the MHRA has issued interim guidance to the NHS not to vaccinate in principle “patients with severe allergies”. It is likely that the restriction on indications now imposed by the MHRA will result in significantly fewer patients being able to receive the vaccine.

Further adverse reaction reports can be expected from the U.S. Food and Drug Administration’s (FDA) Emergency Use Authorization (EUA) for BNT162b2 granted on December 11, 2020, as very large vaccination numbers are now likely to be achieved rapidly.

## Allergic reactions to vaccines

Allergic reactions to vaccines are very rare, occurring at 1 per 1,000,000 to 30 per 100,000 vaccinations [2–6].

Vaccine components known to cause allergic reactions include residues of animal proteins, antimicrobial agents, preservatives, stabilizers, and adjuvants in addition to the active component of the vaccine (the actual antigen) that elicit the immune response [2–6]. Individual vaccine components associated with causing vaccine anaphylaxis include chicken egg protein, gelatin, cow’s milk proteins, and other additives and trace compounds left over from the manufacturing process, in addition to latex components from the sealing plugs in multiple vaccine vials [3–6].

## The vaccine BNT162b2

BNT162b2 is “temporarily licensed” in the United Kingdom for active immunization to prevent COVID-19 disease caused by the SARS-CoV‑2 virus in persons 16 years of age and older [7].

The vaccine is administered intramuscularly in two doses of 0.3 ml each, 21 days apart. Thawed COVID-19 mRNA vaccine BNT162b2 requires dilution in its original vial (0.45 ml) with 1.8 ml of unpreserved sodium chloride 0.9% solution for injection, prior to withdrawing a 0.3 ml dose [7, 8].

The listed excipients in BNT162b2 are ALC-0315 ((4-hydroxybutyl)azanediyl)bis(hexane‑6,1‑diyl)bis(2-hexyl decanoate), ALC-0159 (2-((polyethylene glycol)-2000)-N,N-ditetradecylacetamide), 1,2-distearoyl-sn-glycero-3-phosphocholine, cholesterol, potassium chloride, potassium dihydrogen phosphate, sodium chloride, disodium hydrogen phosphate dihydrate, sucrose, and water for injection [7, 8].

One vial (0.45 ml) contains five doses of 30 µg of highly purified, single-stranded, 5′-capped mRNA (BNT162b2 RNA) produced by cell-free in vitro transcription on an appropriate DNA template and encoding the viral spike(S) protein of SARS-CoV‑2 [7, 8].

This mRNA is embedded in lipid nanoparticles. mRNA is rapidly degraded by ribonucleases, readily taken up by mononuclear phagocytes, and has poor ability to penetrate cell membranes due to its negative electrical charge and high molecular weight. Therefore, mRNA requires a protective envelope for use as a vaccine. In the BioNTech vaccine, lipid-based nanoparticles (LNPs) are used as nonviral vectors for this purpose, containing cationic lipids that coat the polyanionic mRNA with its tertiary or quaternary amines, complemented by zwitterionic lipids that mimic cell membrane phospholipids, and cholesterol that stabilizes the lipid bilayer of the nanoparticle. Finally, polyethylene glycol (PEG)-modified lipids enable the assembly of a hydrate shell, increasing the solubility of the LNPs.

PEG or macrogol is a polyether compound commonly used as an additive in cosmetics, pharmaceuticals, and also in foods [9]. PEG exists in molecular weight types ranging from 200 g/mol to 10,000,000 g/mol and allergic reactions have been reported after its use in a variety of drugs and cosmetic products [10, 11]. There is cross-reactivity to polysorbate 80 due to common chemical motifs [12, 13]. Allergic reactions to PEG are probably diagnosed too rarely, so PEG is also considered a “hidden” allergen [11]. Severe allergic reactions have been described in diagnostic skin testing, so this should only be performed in allergy-specialized centers according to published standard regimens.

In the pivotal phase III clinical trial, the BNT162b2 vaccine was generally well tolerated [8]. A total of 43,548 participants were randomized, of whom 43,448 received one injection, 21,720 with BNT162b2 and 21,728 with placebo 18,556 received a second dose of BNT162b2 [8]. The most common adverse reactions were local reactions at the injection site (84.7%), fatigue (62.8%), headache (55.1%), muscle pain (38.3%), chills (31.9%), joint pain (23.6%), fever (14.2%) [7, 8]. Most reactions were mild to moderate in severity. Severe adverse reactions occurred in up to 4.6% and were more common after the second dose and less common in adults > 55 years of age. Lymphadenopathy was reported in 0.3%. Systemic adverse reactions were usually mild or moderate in severity, generally occurring the day after vaccination and lasting one to two days thereafter [7, 8]. The occurrence of allergic reaction was reported in similar numbers in both the vaccine-verum and placebo groups (0.63% vs. 0.51%) [7, 8]. However, this large clinical trial, used to support vaccine approval by the MHRA and FDA, excluded individuals with a “history of a severe adverse reaction related to the vaccine and/or a severe allergic reaction (e.g., anaphylaxis) to a component of the study medication” [7, 8]. Based on this the UK package insert based on this states that the vaccine should not be administered to individuals who are allergic to the active ingredient or any of the other ingredients listed. In this respect, the patient information complies with the exclusion criteria of the clinical trial.

## Evaluation and outlook

Vaccines are recognized as one of the most effective public health interventions. The primary goal of vaccination programs is the protection of the vaccinated individuals. In addition, in many cases they also aim to protect unvaccinated individuals. The ultimate goal is creating herd immunity, i.e., resistance to the spread of a contagious disease in a population, which occurs when a sufficiently high proportion of individuals are immune to that disease, especially through vaccination [14].

The development of such herd immunity requires vaccination rates of > 60% of the total population [14].

However, to achieve effective individual immunization of > 95% with BNT162b2, a second vaccination (“booster”) is necessary. This repeated administration of a foreign protein (antigen) necessitates a careful allergological history before each application and diagnostic clarification, if necessary, and risk–benefit assessment before each injection.

Against this background we state that

- Patients and people who are to receive a vaccination against COVID-19 must also be regularly informed about possible severe allergic/anaphylactic reactions and questioned with regard to such incidents in the past.
- Allergic reactions to additives, in particular PEG and cross-reactive PEG analogues, must be systematically queried in order to identify patients at risk.
- In suspected cases, allergological clarification (skin prick test, laboratory diagnostics) and consultation of an allergist should be carried out.
- Personnel performing vaccination against COVID-19 must always be prepared for the possibility of severe allergic/anaphylactic reactions and vaccination teams and vaccination centers should be trained in the treatment of anaphylaxis according to the recommendations of the current AWMF guideline on the acute therapy and management of anaphylaxis [15].
- It will be important to understand the specific cause of the two reported severe allergic reactions and the medical history of the individuals involved so that any risks of allergic reactions can be more accurately defined and, if possible, circumvented.
- Current authority guidelines in the UK exclude patients with severe allergies from vaccination with BNT162b2.
- More precise definitions of the type, cause, and severity of severe allergic reactions are needed because, given the high incidence of patients with “severe” allergies (a significant proportion of the total population in Europe and the US, depending on the definition), excluding all such patients from vaccination could have a significant impact on achieving the goal of herd immunity. On the other hand, a more precise definition (e.g., “anaphylaxis-prone patients”) would suggest only 1–3% of the population for whom vaccination would be impossible or only possible with special protective measures.
- More data need to be collected from both clinical trials and clinical practice that will improve our knowledge of the safety profile of COVID-19 vaccines, particularly with regard to severe allergic reactions.

Severe allergic reactions to vaccines are rare but can be life-threatening, and it is prudent to raise awareness of this hazard among vaccination teams and to take adequate precautions while more experience is gained with this new vaccine. Patients with a history of severe allergic reactions may be vaccinated with adequate medication and by physicians experienced in the management of anaphylactic reactions if the ongoing investigation in the UK allows this recommendation. PEG is currently considered to have most likely triggered the severe allergic reactions in the 2 affected patients in the UK, but further investigations are awaited.

If confirmed, it would only be necessary to exclude patients with known allergic reactions to PEG, PEG analogs, and other additives from vaccination with BNT162b2, but not all patients with a history of severe allergic reactions, which would significantly expand the pool of potentially vaccinatable individuals.

For BNT162b2, safety monitoring will continue for 2 years after administration of the second vaccine dose within the study [8].

## Abbreviations

EUA: Emergency Use Authorization
FDA: Food and Drug Administration
LNP: Lipid-based nano particles
MHRA: Medicines and Healthcare products Regulatory Agency
NHS: National Health Service
PEG: Polyethylenglycol
SmPC: Summary of Product Characteristics
S‑Protein: Spike-protein
